# PD-L1 produced by HaCaT cells under polyinosinic-polycytidylic acid stimulation inhibits melanin production by B16F10 cells

**DOI:** 10.1371/journal.pone.0233448

**Published:** 2020-05-21

**Authors:** Minhwa Park, So-Youn Woo, Kyung-Ah Cho, Min-Sun Cho, Kyung Ho Lee

**Affiliations:** 1 Department of Microbiology, College of Medicine, Ewha Womans University, Seoul, Korea; 2 Department of Pathology, College of Medicine, Ewha Womans University, Seoul, Korea; 3 Department of Dermatology, College of Medicine, The Catholic University of Korea, Bucheon-si, Korea; Medical University of Gdańsk, POLAND

## Abstract

Skin forms a physical barrier that protects the body against outside agents. The deepest layer of the skin, the stratum basale, contains two cell types: agent-sensing keratinocytes, and melanin-producing melanocytes. Keratinocytes can sense both harmless commensal organisms and harmful pathogens via Toll-like receptors (TLRs), and keratinocytes subsequently drive immune responses. Activation of TLR3 is required for barrier repair because it stimulates essential genes, including tight junction genes, and inflammatory cytokines. Within the basal layer of the skin, resident melanocytes use their dendritic processes to connect with approximately 30–40 neighboring keratinocytes. Most studies have focused on the transfer of melanin-synthesizing melanosomes from melanocytes to keratinocytes, but the potential regulation of melanogenesis by soluble factor(s) produced by keratinocytes remains to be explored. Studying such regulation *in vivo* is challenging because of the keratinocyte:melanocyte ratio in the epidermis and the location of the cells within the skin. Therefore, in this study, we investigated whether keratinocytes affected melanocyte melanogenesis *in vitro* under normal or inflammatory conditions. We found that polyinosinic-polycytidylic acid [poly(I:C)] stimulation induced PD-L1 secretion from HaCaT cells and that poly(I:C)-induced PD-L1 inhibited melanin production by B16F10 cells. These data provide key evidence that keratinocytes can alter melanocyte melanogenesis via the production of soluble factors under inflammatory conditions.

## Introduction

The skin, which acts as a physical barrier to protect the body against external chemical, physical, and microbiological agents, has three main layers: the epidermis, the dermis, and hypodermis including adipose tissue [1]. The epidermis of thin skin consists of four layers: stratum basale, stratum spinosum, stratum granulosum, and stratum corneum. The stratum basale, which is the deepest layer, contains undifferentiated keratinocytes and melanocytes. Undifferentiated keratinocytes move up from the stratum basale into the stratum spinosum where they begin to differentiate, adopting a polygonal morphology and initiating keratin synthesis. The keratinocytes continue to differentiate as they migrate to the skin surface. Keratinocytes within the stratum granulosum release keratin proteins and lipids. Keratinocytes in the stratum corneum, the outermost layer of the epidermis, stack as multiple layers and function as a physical barrier [2]. Keratinocytes sense chemical, physical, and microbial agents via distinct toll-like receptors (TLRs) that subsequently drive immune responses. Among these TLRs, TLR3 recognizes double-stranded RNA from viruses and also senses non-coding RNA from UV-irradiated epidermal keratinocytes [3]. In fact, TLR3 is the primary sensor of UV exposure and produces an inflammatory response [4]; specifically, following UV irradiation, TLR3 initiates a signaling cascade entailing Toll/Interleukin-1 receptor (TIR) domain-containing signaling interferon-β activation, subsequent activation of nuclear factor-κB and activator protein 1 (AP-1), and AP-1-mediated activation of the mitogen-activated protein kinases pathway via IκB kinase complex activation.

A common complication of skin inflammation, be it from endogenous inflammation, external injury, or cutaneous procedures such as laser therapy, is post-inflammatory hyperpigmentation (PIH). PIH appears locally in previous areas of inflammation, and pigmentation intensity is determined by inherent skin color, severity of inflammation, degree of dermoepidermal junction damage, and stability of melanocytes. Skin discoloration resulting from previous inflammation depends on the number of melanocytes and their functional activity [5]. Melanocytes reside in basal layer of the skin and use their dendritic processes to connect with approximately 30–40 neighboring keratinocytes. Mature melanocytes generate a special organelle called the melanosome, which contains the enzymes responsible for melanin synthesis, such as tyrosinase related protein 1 (TYRP1), transient receptor potential cation channel subfamily M member 1 (TRPM1), and tyrosinase [6]. Mature melanocytes can transfer melanosomes into keratinocytes via their dendritic processes in a phagocytic process promoted by protease-activated receptor-2 [7, 8].

Most studies on melanogenesis have focused on the transfer of melanosomes from melanocytes to keratinocytes. For instance, melanin synthesis by melanocytes increases following UV radiation or mechanical irritation and is associated with increased melanosome transfer to keratinocytes [9]. Separately, Yamasaki *et al*. reported that TLR3 stimulation enhances melanogenesis and melanosome transfer in human melanocytes [10] and also melanosome uptake by keratinocytes [11]. Although they observed that treatment with polyinosinic-polycytidylic acid [poly(I:C)] enhanced melanosome transfer from melanocyte to keratinocytes in a co-culture system via the Rho-family GTPases RHOA and CDC42, it remains unclear if melanocyte melanogenesis is regulated by a soluble factor produced by keratinocytes under inflammatory conditions.

We previously reported that poly(I:C) attenuates imiquimod-induced skin inflammation in mice by increasing cutaneous programmed death-ligand 1(PD-L1) expression and reducing interleukin (IL)-36gamma expression in human HaCaT keratinocytes in the presence of IL-17 [12]. In this study, we investigated whether HaCaT cells affect melanogenesis in B16F10 melanoma cells under TLR3 stimulation and if the effect is mediated by a soluble factor.

## Materials and methods

### Cell culture

Human HaCaT cells and B16F10 murine melanoma cells (Korea Cell Line Bank #80008, Seoul, Korea) were cultured in Dulbecco’s Modified Eagle Medium (DMEM, Welgene, Gyeongsan, Korea) supplemented with 10% fetal bovine serum (FBS, Welgene) and 1% penicillin/streptomycin (PAA Laboratories, Little, UK) at 37°C in 5% CO_2_. Cells were subcultured every 2–3 days. For poly(I:C) stimulation, HaCaT and B16F10 cells were cultured for 6 h in 6-well plates in DMEM containing 10% FBS. The cells were then changed into serum-free DMEM and were subsequently treated overnight with poly(I:C) (Sigma-Aldrich, St. Louis, MO, USA) at a final concentration of 20 μg/mL.

### Co-culture

HaCaT cells (5 × 10^5^) were cultured in the presence or absence of poly(I:C) in the upper chamber (0.4 μm pore diameter) of a Transwell plate (Corning Inc., Corning, MA, USA) for 24 h. Starting 18 h after HaCaT culturing began, B16F10 cells (1.5 × 10^6^) were cultured in a 24-well cell culture plate (Corning Inc.) for 6 h. To initiate co-culturing, B16F10 cells were changed into serum-free DMEM, and the HaCaT-containing Transwell insert was moved into the B16F10-seeded plate. After 6 h, 12 h, and 24 h of co-culturing, the B16F10 cells and the culture medium (CM) were collected for RNA and protein extraction. For targeted PD-L1 experiments, B16F10 cells were treated with recombinant human PD-L1 (rPD-L1, Peprotech, Rocky Hill, NJ, USA) or LEAF^™^ purified anti-human PD-L1 antibody (29E.2A3; BioLegend, San Diego, CA, USA), stored at -20°C, at a final concentration of 10 μg/mL.

### MTT assays

B16F10 cells (5 × 10^4^ cells) were cultured for 6 h in a 96-well flat-bottom plate in DMEM supplemented with 10% FBS and 1% penicillin/streptomycin. After being changed into serum-free DMEM, cells were treated with 20 μg/mL poly(I:C) for 24 h, followed by 20 μL thiazolyl blue tetrazolium bromide solution (MTT, 5 mg/mL in phosphate-buffered saline (PBS), Sigma-Aldrich) was added to each well and the cells were incubated for 4 h at 37°C. The MTT solution was removed, then 200 μL dimethyl sulfoxide (DMSO, Merck, Kenilworth, NJ, USA) was added to each well and the plate was shaken for 15 min to solubilize the formazan. Absorption at 570 nm was measured with an enzyme-linked immunosorbent assay (ELISA) reader (SpectraMax versa; Molecular Devices, San Jose, CA, USA).

### Immunoblot

For intracellular proteins, B16F10 and HaCaT cells were lysed with RIPA buffer (1% Triton X-100, 150 mM NaCl, 20 mM Tris-HCl, pH 7.5) containing protease inhibitors (Sigma-Aldrich) and 200 mM sodium orthovanadate (Na_3_VO_4_, Sigma-Aldrich). For secreted proteins, CM from B16F10 and HaCaT cultures were collected by centrifugation (400 × g for 5 min) and filtered through 0.22 μm filters. Then, 500 μL CM samples were applied to Amicon Ultra-3 kDa filter devices (Millipore, Darmstadt, Germany) and centrifuged at 14,000 × g for 20 min to obtain concentrated CM. To detect secreted PD-L1, concentrated CM were subjected to 10% sodium dodecyl sulfate-polyacrylamide gel electrophoresis (SDS-PAGE) and immunoblot with anti-rabbit PD-L1 primary antibody (rabbit polyclonal, Santa Cruz Biotechnology, Santa Cruz, CA, USA) overnight at 4°C, followed by incubation with HRP-conjugated secondary antibody for 1 h at room temperature. After washing three times, the membranes were developed using ECL solution (GE Healthcare Life Sciences, Pittsburgh, PA, USA), and protein bands were detected using a LAS3000 system (Fujifilm, Tokyo, Japan).

### Flow cytometry

B16F10 cells were incubated with PE-conjugated anti-mouse CD279 antibody (29F.1A12, Rat IgG_2a_, BioLegend) for 30 min on ice. Isotype (PE-conjugated anti-rat IgG_2a_κ, BD Pharmingen, San Diego, CA, USA) stained cells were used as control. Cells were centrifuged at 400 × g for 5 min at RT and fixed with 1% paraformaldehyde in FACS buffer (0.5% FBS in phosphate buffered saline). Cells were detected using a FACSCalibur (BD Biosciences, Franklin Lakes, NJ, USA) and analyzed with Cell Quest software (BD Biosciences).

### Real-time PCR

Total RNA was isolated from B16F10 and HaCaT cells using TRISure (Bioline, London, UK) in accordance with the manufacturer’s instructions. One microgram of total RNA was transcribed into cDNA using Reverse Transcription Master Premix (ELPIS Biotech, Daejeon, Korea). To quantify the expression of melanoma-specific genes, real-time PCR was conducted using a KAPA SYBR qPCR kit (KAPA Biosystems Inc., Woburn, MA, USA) and an ABI StepOne Plus detection system (Applied Biosystems, Foster City, CA, USA). All gene expression values were normalized to the expression of the *Gapdh* housekeeping gene. Real-time PCR was performed using the following primers: mouse microphthalmia-associated transcription factor (*MITF*) (126 bp), 5’- CAAATGGCAAATACGTTACCCG-3’ (forward) and 5’-CTCCCTTTTTATGTTGGGAAGGT -3’ (reverse); mouse *Tyr* (107 bp), 5’- CTCTGGGCTTAGCAGTAGGC -3’ (forward) and 5’- GCAAGCTGTGGTAGTCGTCT -3’ (reverse); and mouse *Gapdh* (95 bp), 5’-AGGTCGGTGTGAACGGATTTG -3’ (forward) and 5’-GGGGTCGTTGATGGCAACA -3’ (reverse).

### Quantification of melanin content

B16F10 cells (1×10^5^ cells/well) were plated and cultured in a 12-well plate overnight. After rinsing, cells were incubated with serum-free, phenol red-free DMEM for different periods of time. Cells and CM were collected by centrifugation for 400 × g, 10 min. CM were filtered through 0.22 μm filters. Cells were homogenized in 1 N sodium hydroxide (Duksan, Seoul, Korea) in 10% DMSO, and cell lysates were incubated in a 60°C water bath for 1 h. Cell lysates and CM were transferred to a 96-well flat-bottom plate and absorbance was measured at 490 nm using an ELISA reader to assess melanin content. Absorbance values were converted to amount melanin using a standard curve generated with synthetic melanin (Sigma-Aldrich). Melanin content was normalized to the total protein content of each sample, as follows. Total protein in cell lysates and CM was quantified by mixing 20 μL sample with bicinchoninic acid assay (BCA) reagent (Thermo Fisher Scientific, Waltham, MA, USA) and incubating at 37°C for 30 min. Absorbance at 562 nm was then measured using an ELISA reader. Normalized intracellular and secreted melanin content was calculated by dividing the measured melanin concentration by the total protein concentration.

### Statistical analysis

Data are presented as mean ± standard deviation (SD). Statistically significant differences between groups were assessed using one-way or two-way analysis of variance (ANOVA) followed by the Tukey-Kramer multiple comparison test. A P value < 0.05 was considered significant. All statistical analyses were performed using GraphPad Prism Software v6.04 (Graph Pad Software Inc., San Diego, CA, USA).

## Results

### Poly(I:C) stimulation induces PD-L1 secretion by HaCaT cells

**Fig 1:**
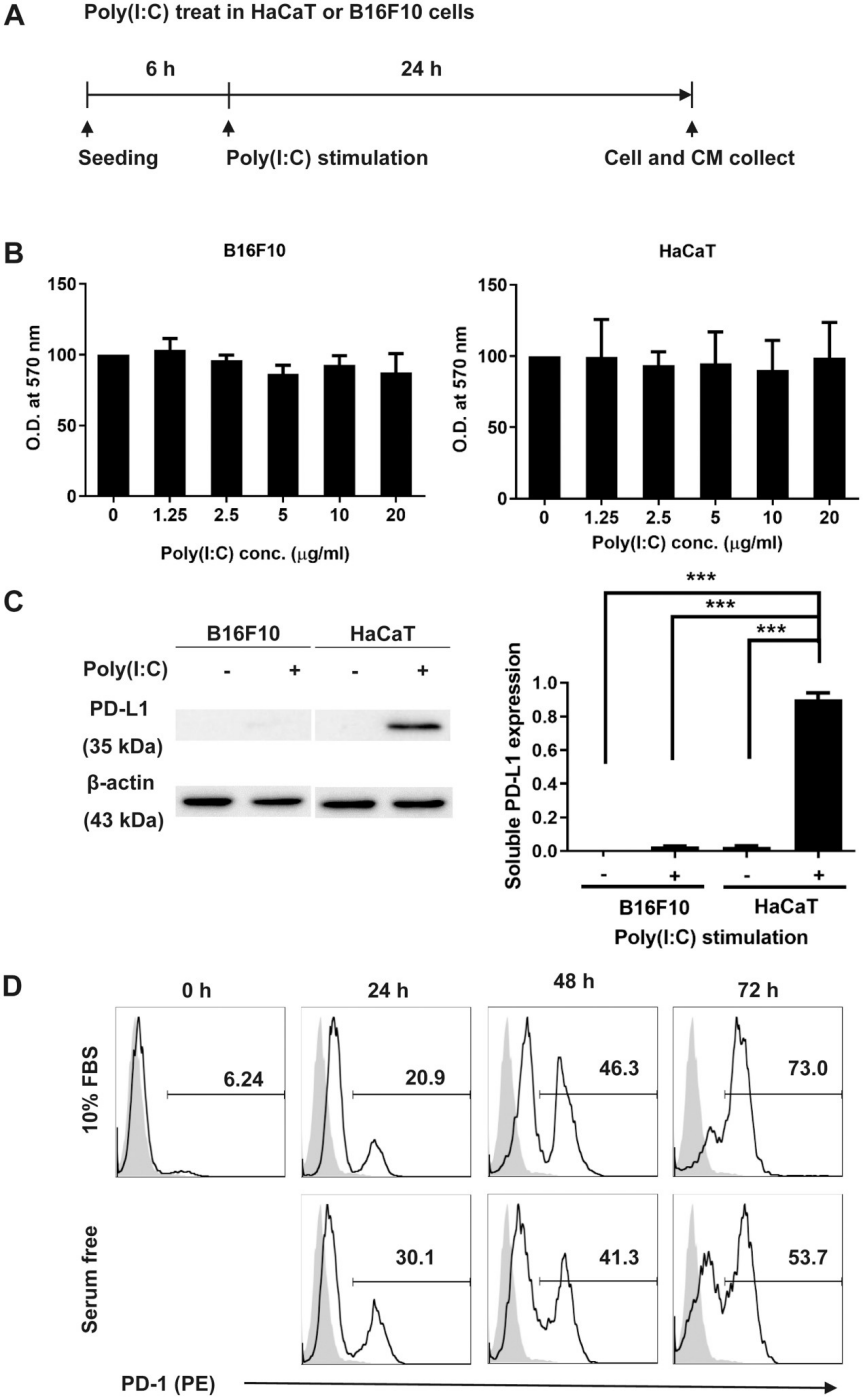
Poly(I:C) stimulation increases PD-L1 secretion from HaCaT cells. (A) HaCaT or B16F10 cells and CM were collected following incubation with 20 ng/mL poly(I:C) for 24 h. (B) Cell viability was measured by MTT assay. (C) PD-L1 levels in CM from B16F10 and HaCaT cultures, assessed by immunoblotting. PD-L1 levels were normalized to β-actin levels using pixel densities. (D) B16F10 cells were cultured in DMEM with or without FBS for ≤ 72 h. Data are presented as mean ± SD and were analyzed by one-way ANOVA (*** *P* < 0.001).

To analyze the effect of poly(I:C) stimulation on metabolic activity of cells, we performed MTT assays on B16F10 and HaCaT cells. After being cultured in DMEM with 10% FBS for 6 h, cells were stimulated with poly(I:C) in serum-free DMEM (Fig 1A). As shown in Fig 1B, poly(I:C) stimulation did not affect the viability of B16F10 cells or HaCaT cells. To assess the level of PD-L1 expression in B16F10 or HaCaT cells after poly(I:C) stimulation, we measured PD-L1 secretion from poly(I:C)-treated B16F10 and HaCaT cells. We found that poly(I:C)-stimulated HaCaT cells expressed significantly higher levels of PD-L1 than did the other cells (Fig 1C). Next, we examined expression of the PD-L1 receptor, programmed cell death protein 1 (PD-1), on the surface of B16F10 cells using flow cytometry. We found that B16F10 PD-1 expression increased over time, with PD-1 surface expression increasing by 72 h in DMEM both with and without 10% FBS (Fig 1D). Therefore, we concluded that B16F10 expressed PD-1.

### Co-culture of poly(I:C)-stimulated HaCaT cells and B16F10 cells leads to decreased expression of melanogenesis-related genes and decreased melanin synthesis

**Fig 2:**
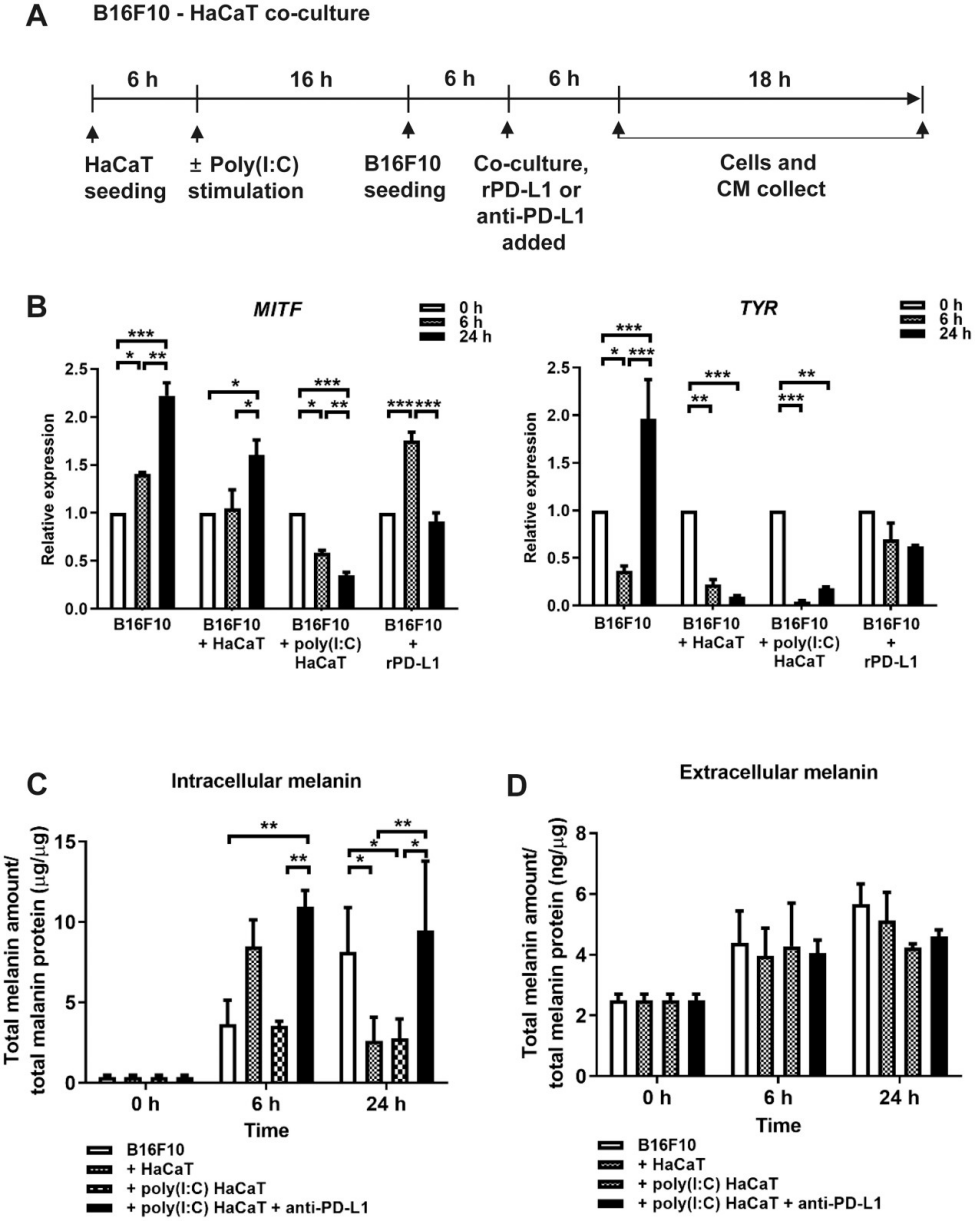
Analysis of melanogenesis-related gene expression and melanin content. (A) HaCaT cells were cultured for 6 h in a Transwell insert and then treated with 20 ng/mL of poly(I:C) for 16 h. Separately, B16F10 cells were cultured for 6 h, and, after changing the B16F10 cells into serum-free DMEM, the HaCaT-incubated Transwell insert was moved into the B16F10-cultured plate. After 6 h and 24 h of co-culture, the B16F10 cells and CM were collected for RT-PCR analysis and quantification of melanin content. (B) Expression of *MITF* and *Tyr* were assessed by real-time PCR using total RNA from B16F10 cells. Select cells were treated with rPD-L1 (10 μg/mL) for indicated periods of time (C) B16F10 cells and (D) CM were collected and centrifuged for 10 min. HaCaT cells were incubated for indicated periods of time with anti-PD-L1 antibody (10 μg/mL). Cells were lysed with 1 N sodium hydroxide in 10% DMSO in a 60°C water bath. To measure melanin content, absorbances of cell lysates and CM were detected at 490 nm using an ELISA reader. Data are presented as mean ± SD and were analyzed by one-way ANOVA (* *P* < 0.05, ** *P* < 0.01, and *** *P* < 0.001).

Next, we examined the effect of poly(I:C)-stimulated HaCaT cells on melanogenesis in B16F10 cells. We used a Transwell insert to co-culture either unstimulated or poly(I:C)-stimulated HaCaT cells with B16F10 cells for 6 and 24 h, at which point B16F10 cells and CM were collected for analysis by quantitative real-time PCR (Fig 2A). Also, to determine the direct effect of PD-L1 on B16F10 cells, we compared B16F10 melanogenesis in the presence and absence of exogenous rhPD-1 (Fig 2B). Expression of *MITF* increased significantly in B16F10 cells after 24 h of co-culture with unstimulated HaCaT cells. However, when co-cultured with poly(I:C)-stimulated HaCaT cells or cultured in the presence of rhPD-1, B16F10 *MITF* expression was significantly reduced after 24 h. Expression of the tyrosinase-encoding *Tyr* gene significantly increased after 24 h in B16F10 but decreased in B16F10 cells co-cultured with either unstimulated or poly(I:C)-stimulated HaCaT cells for 24 h. Treatment with rPD-L1 did not substantially affect *Tyr* expression after 6 or 24 h. In summary, both poly(I:C)-stimulated HaCaT cells and rPD-L1 inhibited expression of the melanin synthesis-associated genes *MITF* and *Tyr* in B16F10 cells.

Next, to enable comparison of melanin production between experimental groups, we quantified intracellular and secreted melanin in B16F10 cells and CM, respectively (Fig 2C and 2D). The intracellular melanin content in B16F10 cells increased significantly after 24 h (8.13 ± 2.78). In B16F10 cells co-cultured with unstimulated HaCaT cells (2.62 ± 1.46) or poly(I:C)-stimulated HaCaT cells (2.76 ± 1.21), the melanin content decreased significantly after 24 h. However, in B16F10 cells treated with anti-PD-L1 antibody, which neutralized the PD-L1 secreted by poly(I:C)-stimulated HaCaT cells, melanin content increased significantly after 6 h (9.48 ± 4.32). However, the amount of secreted melanin was very small (approximately 2.50–5.66 ng secreted melanin per μg total protein) compared to the amount of intracellular melanin (approximately 0.36–10.97 μg intracellular melanin per μg total protein). Overall, the results of this study demonstrate that poly(I:C) stimulation induces PD-L1 secretion by HaCaT cells, and HaCaT-produced PD-L1 inhibits expression of melanogenesis-related genes and melanin production in B16F10 cells.

## Discussion

We previously reported a relationship between keratinocyte PD-L1 expression and skin inflammation [12]. Our report showed that PD-L1 from poly(I:C)-stimulated keratinocytes dampens IMQ-induced skin inflammation by inhibiting Th17 cell differentiation [13] and blocks signaling via the IL-17 receptor [12]. The PD-1/PD-L1 pathway represents an immune checkpoint because PD-1 (also known as CD279) is expressed on activated T cells, and interaction between PD-1 and its ligand, PD-L1 or PD-L2, inhibits TCR activation. Immune checkpoint blockade using chemical inhibitors has attracted considerable attention recently for tumors that induce immune suppression [14], as T-cell inhibition by co-stimulatory immune checkpoint (e.g., CTLA-4/CD80 binding) leads to T-cell anergy [15].

Melanocytes differentiate from neural crest cells via Wnt1/3a signaling [16, 17]. Immature melanocytes move to specific tissues (e.g., skin, hair, and iris) where they proliferate or mature. Development, differentiation, and maturation of melanocytes is regulated primarily by MITF, Kit, Snail/Slug, Sox10, and endothelins [18]. MITF is the only one transcription activator to regulate transcription of melanogenesis-controlling enzymes, including tyrosinase (TYR), tyrosinase-related protein 2 (TRP-2), and tyrosinase-related protein 1 (TRP-1). Upstream of MITF are Wnt/beta-catenin pathway effector LEF-1 and cAMP pathway effector cAMP response element binding (CREB) to transactivate MITF gene in melanocytes [19]. The interaction of stem cell factor (SCF) from keratinocytes with its receptor Kit expressed on melanocytes is important in melanocyte development, migration and survival [20, 21]. External stimuli, such as UV radiation, induce the melanocortin-1 receptor (MC1R) in melanocytes and induce keratinocyte production of alpha-melanocyte-stimulating hormone, which subsequently activates MC1R in melanocytes to initiate melanin production [22].

In this study we found that poly(I:C) stimulated keratinocytes affected melanogenesis in B16F10 cells, especially via down-regulation of MITF expression. Because MITF overexpression is related to oncogenic potential as “lineage addiction,” which allows melanoma cells retain lineage-persistent tumor and promoting cell survival and proliferation [23], poly(I:C) stimulated keratinocytes possibly can suppress the growth of melanoma cells via MIFT down-regulation by PD-L1/PD-1 interaction. In addition, PD-L1 from poly(I:C) treated keratinocytes might reverse the inhibitory immune regulation mechanism of PD-1 on T cells in tumor—T cell interactions. It was reported that nanoplexed form of poly(I:C) injection to B16F10 cells-derived tumor showed antitumor activity by increasing tumor-specific CD8+ T cells and type I interferon pathway [24].

Because melanin is insoluble in all solvents and complexed with other intrinsic proteins, there are diverse analytical methods for melanin quantification. For example, electron paramagnetic resonance (EPR) spectrometry [25], pyrrole-2,3,5-tricarboxylic acid (PTCA) or aminohydroxyphenylalanine (AHP) detection by high performance liquid chromatography (HPLC) [26], and absorption spectroscopy [10]. Although EPR spectrometry and HPLC are direct method to identify unknown natural pigment, such as from malignant lung cancer tissue, absorption spectroscopy is the most widely used due to simple procedure and does not require skilled operator or assay machine. The caveats of absorption spectroscopy for melanin is not specific due to all components of cells are dissolved with strong NaOH solution at high temperature. However, we chose to use NaOH lysis method to compare melanin content in B16F10 melanoma cell lines under PD-L1 effect.

In summary, here we show that PD-L1 from poly(I:C)-treated HaCaT cells induced downregulation of melanogenesis-related genes and melanin production in B16F10 cells. This suggests that keratinocytes negatively affect melanogenesis via the PD-L1/PD-1 pathway under inflammatory conditions, such as during TLR3 stimulation. Although studies examining the negative regulation of melanogenesis human normal melanocyte *in vitro* as well as *in vivo* model are needed, our results provide insight into potential mechanisms underlying normal and abnormal melanin production, such as in PIH.

## Supporting information

S1 Raw images (PDF)
