# Supplementary material for: PD-L1 produced by HaCaT cells under polyinosinic-polycytidylic acid stimulation inhibits melanin production by B16F10 cells
Source: PLoS One. 2020 May 21;15(5):e0233448. doi: 10.1371/journal.pone.0233448 (PMC7241723; doi:10.1371/journal.pone.0233448)
Supplement: S1 Raw images — (PDF) [file pone.0233448.s001.pdf]

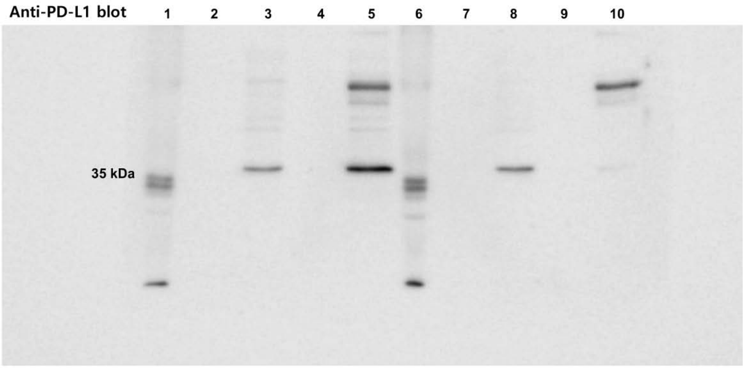

1	Marker	6	Marker
2	HaCaT - media change	7	B16F10 - media change
3	HaCaT - Poly(I:C) Tx, media change	8	B16F10 - Poly(I:C) Tx, media change
4	HaCaT	9	B16F10
5	HaCaT - Poly(I:C) Tx	10	B16F10 - Poly(I:C) Tx

Anti-β-actin blot

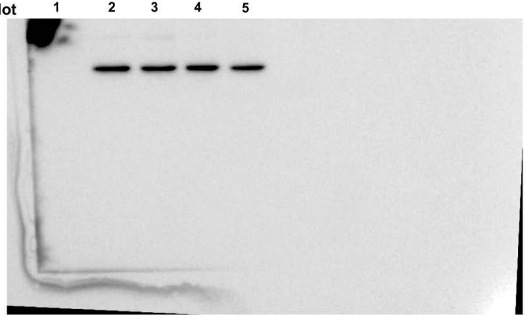

1	Marker
2	B16F10
3	B16F10 - Poly(I:C) Tx
4	HaCaT
5	HaCaT - Poly(I:C) Tx
